# Platinum sensitivity and CD133 expression as risk and prognostic predictors of central nervous system metastases in patients with epithelial ovarian cancer

**DOI:** 10.1186/1471-2407-14-829

**Published:** 2014-11-17

**Authors:** Bo-lin Liu, Shu-juan Liu, Andrius Baskys, Hong Cheng, Ying Han, Chao Xie, Hui Song, Jia Li, Xiao-yan Xin

**Affiliations:** Department of Neurosurgery, Xijing Institute of Clinical Neuroscience, Xijing Hospital, Fourth Military Medical University, Xi’an, Shaanxi Province People’s Republic of China; Department of Obstetrics and Gynecology, Xijing Hospital, Fourth Military Medical University, West Changle Road, No.127, Xi’an, 710032 Shaanxi Province People’s Republic of China; Riverside Psychiatric Medical Group and School of Medicine, University of California Riverside, 5887 Brockton Avenue, Ste. B, Riverside, CA 92506 USA; Department of Pathology, Xijing Hospital, Fourth Military Medical University, Xi’an, Shaanxi Province People’s Republic of China; Department of Prosthodontics, School of Stomatology, Fourth Military Medical University, Xi’an, Shaanxi Province People’s Republic of China; Department of Dental Implantology, School of Stomatology, Fourth Military Medical University, Xi’an, Shaanxi Province People’s Republic of China

**Keywords:** Brain metastases, Chemoresistance, Prognosis, Stem cell marker

## Abstract

**Background:**

To characterize prognostic and risk factors of central nervous system (CNS) metastases in patients with epithelial ovarian cancer (EOC).

**Methods:**

A retrospective analysis of Xijing Hospital electronic medical records was conducted to identify patients with pathologically confirmed EOC and CNS metastases. In addition to patient demographics, tumor pathology, treatment regimens, and clinical outcomes, we compared putative cancer stem cell marker CD133 expression patterns in primary and metastatic lesions as well as in recurrent EOC with and without CNS metastases.

**Results:**

Among 1366 patients with EOC, metastatic CNS lesions were present in 29 (2.1%) cases. CD133 expression in primary tumor was the only independent risk factor for CNS metastases; whilst the extent of surgical resection of primary EOC and platinum resistance were two independent factors significantly associated with time to CNS metastases. Absence of CD133 expression in primary tumors was significantly associated with high platinum sensitivity in both patient groups with and without CNS metastases. Platinum resistance and CD133 cluster formation in CNS metastases were associated with decreased survival, while multimodal therapy including stereotactic radiosurgery (SRS) for CNS metastases was associated with increased survival following the diagnosis of CNS metastases.

**Conclusions:**

These data suggest that there exist a positive association between CD133 expression in primary EOC, platinum resistance and the increased risk of CNS metastases, as well as a less favorable prognosis of EOC. The absence of CD133 clusters and use of multimodal therapy including SRS could improve the outcome of metastatic lesions. Further investigation is warranted to elucidate the true nature of the association between platinum sensitivity, CD133 expression, and the risk and prognosis of CNS metastases from EOC.

## Background

The estimated incidence of central nervous system (CNS) metastases in patients with epithelial ovarian cancer (EOC) is 1.01% (range from 0.49% - 2.2%) [1]. Recently, an increased incidence of CNS metastases in EOC has been reported [2–4], possibly due to a result of better control of the primary cancer, advances in CNS imaging techniques, and use of platinum-based chemotherapies [4]. Platinum compounds do not pass the blood–brain barrier (BBB), leaving the CNS more vulnerable to the growth of cancer cells [4], and reportedly platinum could damage the BBB facilitating metastatic cancer cell entry [5]. Increasing prevalence of CNS metastases associated with EOC underscores the importance of and the need for a better understanding of this clinical entity. However, in most centers, diagnostic brain imaging is not a routine procedure during the follow-up workup for EOC, and the standard monitor tools such as CA-125 do not reliably predict CNS metastases.

It has been shown that prognostic factors for EOC patients with CNS metastases vary. Thus, a high performance status [6, 7], absence of extracranial lesions accompanying CNS metastases [8, 9], single metastases [7, 8], platinum sensitivity [7], a longer time to develop CNS metastases [10], recursive partitioning analysis class [11], and a multimodal therapy for CNS lesions [9, 11, 12] are often associated with a more favorable prognosis. CD133 (prominin-1), a 5-transmembrane glycoprotein [13] that is a putative marker for cancer stem cells (CSCs) in solid tumors including ovarian cancer, has been thought to define a subpopulation of tumor-initiating cells with enhanced resistance to platinum [14–16]. CD133 expression was shown to be an unfavorable prognostic factor for overall and disease-free survival in patients with ovarian cancer, which is also associated with poor response to platinum-based chemotherapy [17]. However, CD133 expression has not been evaluated in patients with CNS metastases. In addition, as a marker for “stemness”, CD133 is shown to be associated with brain tumor stem cells that play key roles in both brain tumor initiation and recurrence because of their capacity for self-renewal and inherent chemo- and radio-resistance [18]; but limited data are available on its role in tumor metastasis.

In this study we examined possible predictors of CNS metastases associated with EOC, and attempted to define a subgroup of vulnerable patients for whom special attention should be paid when monitoring and managing disease progression and CNS metastases.

## Methods

### Patients

Patient records at Xijing Hospital (Xi’an, People’s Republic of China) between January 2002 and December 2011 were included in the study if they had pathologically confirmed EOC. Patients excluded from the study were those with 1) a past history of malignancy other than EOC, 2) a synchronous primary tumor of other organs and 3) a non-epithelial histologic type of ovarian cancer. Demographic, clinical, and pathologic data related to the primary cancer were obtained from the institution’s medical records database. Patients were divided into platinum sensitive (complete clinical remission with a treatment-free interval >6 months after prior platinum therapy) or platinum resistant (progression or relapse within 6 months) groups [7, 17]. Among all the 1366 patients with EOC, 29 with CNS malignancies were identified. The patients’ demographic and clinical characteristics were reevaluated regarding the presence of CNS metastases. During the study period, there was no established treatment protocol for these patients with CNS metastases whose treatments were retrospectively reviewed. Thirty-one pathology-matched EOC patients with at least 1 relapse of disease but without CNS metastases were used as the control. Approval was obtained from the Institutional Review Board of Xijing Hospital, Fourth Military Medical University to perform this study and to use archived material for research purposes.

### Immunohistochemistry

**Figure 1:**
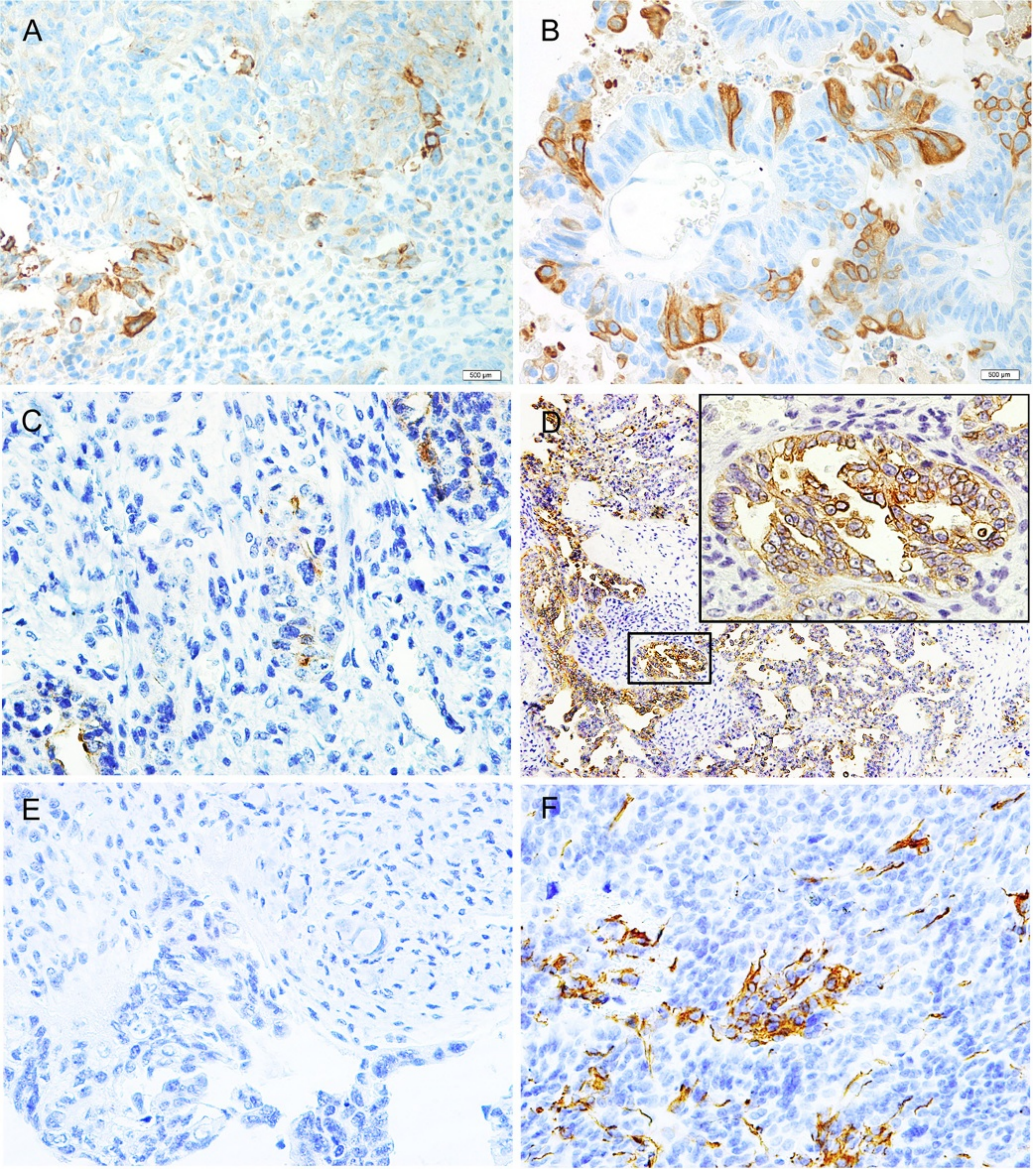
**Representative example of CD133 immunoreactivity pattern in ovarian cancer and CNS metastases (original magnification, x40). (A)** Cell membrane expression in ovarian serous cystadenocarcinoma. **(B)** Cell membrane and cytoplasmic expression in ovarian mucinous cystadenocarcinoma. **(C)** Positive single cell expression pattern in CNS metastases from ovarian cancer. **(D)** Positive cluster formation in CNS metastases from ovarian cancer (original magnification, x10, insert showing higher magnification, x40). **(E)** Negative control. **(F)** Positive control of glioblastoma.

Immunohistochemistry was done as previously described [17]. Briefly, rabbit polyclonal antibody against CD133 (Abcam, Cambridge, UK) was used to detect CD133 expression in the EOC tissues of all patients with (*N* = 29) and without (*N* = 31) CNS metastases and the metastatic CNS tumor tissues obtained during neurosurgery (*N* = 19), using the standard two-step indirect immunohistochemical staining method. We used the glioblastoma tissue as a positive control of CD133 (Figure 1F). Omitting CD133 antibody during the primary antibody incubation served as a negative control (Figure 1E).

Assessment of CD133 expression was done independently by two observers (BLL and HC) blinded to clinicopathological information. Presence of either membrane and/or cytoplasmic staining were considered a positive signal, and the score of each sample was calculated as a mean proportion of positive cells (range, 0-100%) in two continuous sections. For statistical analysis, all cases were divided into CD133- (0% CD133+ tumor cells) and CD133+ (>0% CD133+ tumor cells, i.e. containing at least one CD133+ cell) [17, 19].

### Statistical analysis

The time to diagnosis of CNS metastases was calculated from the time of primary cancer surgery to the time of imaging diagnosis of CNS lesions. Overall survival (OS) after the diagnosis of CNS metastases was calculated from the time of imaging diagnosis to the time of death as a result of any cause. Patients who were alive at the time of the last follow-up (November of 2012) were censored. Probability of survival was estimated using the Kaplan-Meier method. Differences in survival were tested by the log-rank test for univariate comparisons. A multivariate analysis with Cox proportional hazards model was done to establish independent predictor(s) for time to CNS metastases and OS after CNS metastases, whereas a multivariate analysis with binary and multinomial logistic regression was done to establish risk factors for the development of CNS metastases. To test whether frequency distributions differed across categorical variables, the Fisher exact test was used. Statistical significance was set at *P* <0.05, based on *N* = 29 cases, unless indicated otherwise. Statistical analysis was performed using SPSS software (version 16.0, SPSS, Inc., Chicago, IL, USA).

## Results

### Patient characteristics

Of 1366 patients diagnosed with EOC, 29 (2.1%) developed CNS metastases. The major clinical characteristics of these 29 patients at the time of diagnosis of primary cancer are summarized in Table 1. The median age was 57 years (range from 37 to 74 years). All patients but 1 had received initial platinum-based chemotherapy. Demographic and clinical features were not significantly different between the EOC patients with CNS metastases and control group (Table 2).

**Table 1:** **Major clinical characteristics related to primary EOC and its association with CD133 expression**

Parameter	No. of patients (%)			***P***value
	CD133-negative expression	CD133-positive expression	Total	
Age (yrs)				0.272
<60	10 (62.5)	6 (37.5)	16	
> = 60	5 (38.5)	8 (61.5)	13	
FIGO stage				1.000
1,2	2 (66.7)	1 (33.3)	3	
3,4	13 (50.0)	13 (50.0)	26	
Pathology of primary cancer				0.837†
Serous	8 (50.0)	8 (50.0)	16	
Mucinous	2 (100)	0 (0)	2	
Endometrioid	1 (50.0)	1 (50.0)	2	
Clear cell	1 (100)	0 (0)	1	
Mixed epithelial	2 (40.0)	3 (60.0)	5	
Undifferentiated	1 (66.7)	2 (33.3)	3	
Histological grade*				0.640
1,2	3 (60.0)	2 (40.0)	5	
3,	8 (44.4)	10 (55.6)	18	
Extent of surgical resection				0.477
TAH + BSO	13 (56.5)	10 (43.5)	23	
Limited	2 (40.0)	3 (60.0)	5	
Biopsy	0 (0)	1 (100)	1	
Lymph node metastasis				0.812
Yes	9 (50.0)	9 (50.0)	18	
No	6 (54.5)	5 (45.5)	11	
Ascites at the time of primary surgery				0.893
Yes	10 (52.6)	9 (47.4)	19	
No	5 (50.0)	5 (50.0)	10	
Adjuvant therapy				0.397
Chemotherapy	14 (56.0)	11 (44.0)	25	
Chemotherapy + Radiotherapy	1 (66.7)	2 (33.3)	3	
None	0 (0)	1 (100)	1	
Platinum sensitivity				0.006
Sensitive	12 (75.0)	4 (25.0)	16	
Resistant	2 (16.7)	10 (83.3)	12	

*Abbreviations:* *TAH* Total abdominal hysterectomy, *BSO* Bilateral salpingo-oophorectomy.

*Where data were available.

†*P* value was caculated by comparing serous *vs.* non-serous groups.

**Table 2:** **Major clinical characteristics and CD133 expression of EOC patients with** ***vs.*** **without CNS metastases**

Parameter	No. of patients (%)		***P***value
	EOC w/ CNS metastases	EOC w/o CNS metastases	
Age (yrs)			0.599
<60	16 (55.2)	15 (48.4)	
> = 60	13 (44.8)	16 (51.6)	
FIGO stage			0.666
1,2	3 (10.3)	2 (6.5)	
3,4	26 (89.7)	29 (93.5)	
Pathology of primary cancer			0.979†
Serous	16 (55.2)	17 (54.8)	
Mucinous	2 (6.9)	2 (6.5)	
Endometrioid	2 (6.9)	2 (6.5)	
Clear cell	1 (3.4)	2 (6.5)	
Mixed epithelial	5 (17.2)	5 (16.1)	
Undifferentiated	3 (10.3)	3 (9.7)	
Histological grade*			0.724
1,2	5 (21.7)	4 (16.7)	
3,	18 (78.3)	20 (83.3)	
Extent of surgical resection			0.572
TAH + BSO	23 (79.3)	26 (83.9)	
Limited	5 (17.2)	5 (16.1)	
Biopsy	1 (3.4)	0 (0)	
Lymph node metastasis			0.313
Yes	18 (62.1)	23 (74.2)	
No	11 (37.9)	8 (25.8)	
Ascites at the time of primary surgery			0.650
Yes	19 (46.3)	22 (52.6)	
No	10 (53.7)	9 (47.4)	
Adjuvant therapy			0.674
Chemotherapy	25 (86.2)	24 (77.4)	
Chemotherapy + Radiotherapy	3 (10.3)	5 (16.1)	
None	1 (3.4)	2 (6.5)	
Platinum sensitivity			0.516
Sensitive	16 (57.1)	19 (65.5)	
Resistant	12 (42.9)	10 (34.5)	
CD133 expression			0.018
Negative	15 (51.7)	25 (80.6)	
Positive	14 (48.3)	6 (19.4)	

*Abbreviations:* *TAH* Total abdominal hysterectomy, *BSO* Bilateral salpingo-oophorectomy.

*Where data were available.

†*P* value was caculated by comparing serous *vs.* non-serous groups.

Diagnosis of CNS metastases was based on CNS imaging abnormalities in all patients and was pathologically confirmed after neurosurgery in 19 patients. The patient characteristics at the time of diagnosis of CNS metastases are presented in Table 3. The median age at diagnosis of CNS metastases was 59 years (range from 39 to 76 years). Neurological deficits (including motor, sensory or cranial nerve damage), headache (with/without nausea and vomiting) and seizures were the most common neurological symptoms and occurred in 17 patients (58.6%), 12 patients (41.3%), and 5 patients (17.2%), respectively. Less common symptoms included altered mental status, dizziness and speech difficulties. In this cohort, 1 patient (3.4%) had leptomeningeal dissemination, 5 patients (17.2%) had intraperitoneal relapse before the disease metastasized to CNS, and 13 patients (44.8%) developed concurrent metastases to other organs at the time of the diagnosis of intracranial lesions.

**Table 3:** **Major clinical characteristics related to CNS metastases and its association with CD133 expression**

Parameter	No. of patients (%)*						***P***value†	***P***value‡	***P***value§
	CD133-negative expression	CD133-positive expression			NA	Total			
		Total	Single cell	Cluster					
Age at the time of CNS metastasis(yrs)							0.228	0.342	1.000
<60	3 (27.3)	8 (72.7)	2 (18.2)	6 (54.5)	4	15			
> = 60	0 (0)	8 (100.0)	2 (25.0)	6 (75.0)	6	14			
KPS score							1.000	0.869	1.000
> = 80	1 (11.1)	8 (88.9)	2 (22.2)	6 (66.7)	5	14			
<80	2 (20.0)	8 (80.0)	2 (20.0)	6 (60.0)	5	15			
No. of metastases							0.546	0.149	0.118
Single	2 (25.0)	6 (75.0)	3 (37.5)	3 (37.5)	3	11			
Multiple	1 (9.1)	10 (90.9)	1 (9.1)	9 (81.8)	7	18			
Leptomeningeal dissemination							/	/	/
Yes	/	/	/	/	1	1			
No	2	11	2	9	2	15			
Unknown	1	5	2	3	7	13			
Prior cancer relapse before the diagnosis of CNS metastasis							1.000	0.728	0.529
Yes	0 (0)	3 (100.0)	0 (0)	3 (100.0)	2	5			
No	3 (18.8)	13 (81.3)	4 (25.0)	9 (56.3)	8	24			
Presence of extracranial disease							1.000	1.000	1.000
Yes	1 (11.1)	8 (88.9)	2 (22.2)	6 (66.7)	4	13			
No	2 (20.0)	8 (80.0)	2 (20.0)	6 (60.0)	6	16			
CD133 expression in primary ovarian cancer							0.211	0.003	0.019
Negative	3	7	4	3	5	15			
Positive	0	9	0	9	5	14			
Treatment of CNS metastases							0.537^#^	0.521^#^	0.580^#^
WBRT	/	/	/	/	7	7			
Steroids	/	/	/	/	2	2			
Neurosurgery	0 (0)	2 (100.0)	0 (0)	2 (100.0)	/	2			
Neurosurgery + WBRT	1 (16.7)	5 (83.3)	1 (16.7)	4 (66.7)	/	6			
Neurosurgery + WBRT + chemotherapy	0 (0)	4 (100.0)	1 (25.0)	3 (75.0)	/	4			
Neurosurgery + SRS	1 (25.0)	3 (75.0)	1 (25.0)	2 (50.0)	/	4			
Neurosurgery + SRS + chemotherapy	1 (33.3)	2 (66.7)	1 (33.3)	1 (33.3)	/	3			
SRS + chemotherapy	/	/	/	/	1	1			

*Abbreviations:* *NA* Not available, *KPS* Karnofsky performance status, *WBRT* Whole-brain radiation therapy, *SRS* Stereotactic radiosurgery.

*Percentage (%) represented the proportion of each group in 19 patients whose tumor tissues were available.

†*P* values were calculated by comparing CD133-negative *vs.* CD133-positive groups in 19 patients whose tumor tissues were available.

‡*P* values were calculated by comparing CD133-negative *vs.* CD133-positive single cell *vs.* CD133-positive cluster groups in 19 patients whose tumor tissues were available.

§*P* values were calculated by comparing CD133-positive single cell *vs.* CD133-positive cluster groups in 16 patients whose tumor tissues were stained CD133 positive.

^#^ *P* values were calculated by comparing neurosurgery + WBRT +/− chemotherapy *vs.* neurosurgery + SRS +/− chemotherapy groups.

Among the 29 patients, a multimodal approach (combination of at least two treatment modalities including neurosurgery, whole-brain radiation therapy [WBRT], stereotactic radiosurgery [SRS] and chemotherapy) was the main treatment, accounting for 62.1% (18 patients). The remaining patients received a monotherapy of either WBRT, neurosurgery, or steroids (7, 2, and 2 patients respectively). Of the 19 neurosurgeries performed either alone or in combination with other modalities, 9 (47.4%) cases were solitary CNS metastases and 10 (52.6%) cases represented multiple lesions.

### Immunohistochemical study of CD133 in primary EOC and CNS metastases

Expression of CD133 in primary and metastatic tumor tissues was present in the membrane and/or cytoplasm, sometimes with a low level of heterogeneity (Figure 1A-D). CD133+ staining was observed in 14 out of 29 (48.3%) primary EOC samples with CNS metastases, 6 out of 31 (19.4%) EOC samples without CNS metastases and 16 out of 19 (84.2%) CNS metastatic tissue samples. The number of CD133+ tumor cells ranged from 0% to 39% (mean 6%) in primary EOC with CNS metastases, from 0% to 33% (mean 2%) in EOC without CNS metastases, and from 0% to 42% (mean 10%) in the 19 CNS metastatic tissue samples. The distribution of CD133 expression in primary EOC with CNS metastases is summarized in Table 1 according to the clinicopathologic characteristics. Absence of CD133 expression in primary EOC with CNS metastases was associated with a higher platinum sensitivity. Specifically, CD133+ expression was observed in 25.0% (4 of 16) patients with platinum-sensitive disease *vs.* 83.3% (10 of 12) in platinum-resistant disease (*P* = 0.006). Similar finding was observed in recurrent EOC without CNS metastases, with CD133+ expression being detected in 5.2% (1 of 19) platinum-sensitive patients *vs.* 40.0% (4 of 10) platinum-resistant patients (*P* = 0.036). No other association was found between CD133+ expression and the clinicopathologic parameters in either EOC with CNS metastases or control group (data not shown).

Results of CD133 expression analysis in CNS metastatic tissue are shown in Table 3. The expression level was high in CNS metastases, suggesting that the categorization of patients in CD133+ *vs.* CD133- may have been biased considering the possible quantitative effect of CD133+ cells [20]. To reduce the bias, we divided samples into a single cell (Figure 1C) and cluster-type staining based on their topology. A cluster was defined as an aggregation of more than five CD133+ cells [21] and sections with at least one cluster were classified as “cluster + type”. CD133+ cell clusters (Figure 1D) more frequently occurred in CNS metastases (63.2%, 12 of 19 patients) but were relatively uncommon in primary EOC (24.1%, 7 of 29 patients). This difference was significant (*P* = 0.015). However, CD133+ cell cluster formation in CNS metastases was associated with CD133+ expression in primary EOC (*P* = 0.003) (Table 3). There was no correlation between the percentage of CD133+ cells in the metastases and the corresponding primary EOC; but there was a correlation between the CD133+ category (samples with >0% CD133+ cells) in the metastases and primary EOC (Spearman’s rank correlation coefficient, r = 0.706; *P* = 0.001). CD133 expression status was concordant between primary and CNS metastatic sites in 12 patients (63.2%) and no statistically significant difference was observed (kappa = 0.289, *P* = 0.211, Table 4). Of the 7 discordant cases, all had CD133+ expression in CNS metastases but not in primary tumors. We analyzed the difference between the concordant and discordant cases according to clinicopathologic parameters at the time of initial EOC diagnosis and found no differences (data not shown). There was no other association observed between CD133+ expression (or cluster formation) and the clinicopathologic parameters examined (Table 3).

**Table 4:** **CD133 expression in primary EOC and corresponding CNS metastatic sites**

CD133 expression status	No. of CD133- (P)	No. of CD133+ (P)
No. of CD133- (M)	3	0
No. of CD133+ (M)	7*	9

*Abbreviations:* *P* Primary tumors, *M* Corresponding CNS metastatic sites.

*P* value = 0.211.

*Discordant cases.

### Risk factors associated with the development of CNS metastases

As shown in Table 2, CD133+ expression was the only factor associated with an increased risk of CNS metastases in recurrent EOC patients, which was significantly different between EOC patients with and without CNS metastases (*P* = 0.018).

Results of binary logistic regression showed that lymph node metastasis at initial surgery and CD133 expression were significantly associated with an increased risk of CNS metastases (data not shown).

Multivariate logistic regression demonstrated CD133 expression in primary tumor as the only independent risk factor for CNS metastases (HR, 4.72; 95% CI, 1.10-20.41; *P* = 0.037) (Table 5).

**Table 5:** **Multivariate logistic regression for risk of CNS metastases**

Variable	HR (95% CI)	***P***value
Age > = 60 yrs	2.74 (0.60-12.58)	0.195
FIGO stage: 3,4 *vs.* 1,2	2.79 (0.42-18.52)	0.289
Pathology: serous *vs.* non-serous	1.87 (0.42-8.23)	0.409
Surgical resection: TAH + BSO *vs.* Limited and Biopsy	1.39 (0.26-7.59)	0.703
Presence of lymph node metastasis	4.17 (0.94-16.67)	0.053
Presence of ascites	1.84 (0.23-14.71)	0.566
Platinum resistance	4.15 (0.83-20.83)	0.083
CD133 expression	4.72 (1.10-20.41)	0.037

*Abbreviations:* *TAH* Total abdominal hysterectomy, *BSO* Bilateral salpingo-oophorectomy.

### Risk factors associated with shorter times to the diagnosis of CNS metastases

Among the 29 patients with CNS metastasis, the median time to the diagnosis of CNS metastases was 23.5 months (range from 6.2-75.0 months).

A univariate analysis of risk factors associated with a shorter time to CNS metastases is shown in Table 6. Factors including International Federation of Gynecology and Obstetrics (FIGO) stage, extent of surgical resection, lymph node metastasis at initial surgery, platinum sensitivity, and CD133 expression were significantly related to the time of the CNS metastases diagnosis.

**Table 6:** **Univariate analysis for predictors of time to CNS metastases**

Variables	No. of patients (%)	Median time to CNS metastases, mo (95% CI)	***P***value
Age (yrs)			0.056
<60	16 (55.2)	27.3 (17.5-37.1)	
> = 60	13 (44.8)	22.9 (14.4-29.4)	
FIGO stage			0.021
1,2	3 (10.3)	45.1 (10.5-79.7)	
3,4	26 (89.7)	22.7 (18.7-26.7)	
Pathology of primary cancer			0.595
Serous	16 (55.2)	25.7 (16.5-34.9)	
Non-serous	13 (44.8)	22.7 (20.8-24.6)	
Histological grade			0.354
1,2	5 (21.7)	27.7 (18.7-36.7)	
3,	18 (78.3)	21.9 (17.1-26.7)	
Extent of surgical resection			<0.0001*
TAH + BSO	23 (79.3)	27.7 (23.0-32.4)	
Limited	5 (17.2)	16.3 (15.4-17.2)	
Biopsy	1 (3.4)	6.2	
Lymph node metastasis			0.026
No	11 (37.9)	31.7 (24.4-39.0)	
Yes	18 (62.1)	22.0 (21.6-22.4)	
Ascites at the time of primary surgery			0.153
No	10 (34.5)	29.0 (22.8-35.2)	
Yes	19 (65.5)	22.1 (21.2-23.0)	
Adjuvant therapy			0.683†
Chemotherapy	25 (86.2)	25.3 (20.4-30.2)	
Chemotherapy + Radiotherapy	3 (10.3)	21.9 (14.9-28.9)	
None	1 (3.4)	23.5	
Platinum sensitivity			<0.0001
Sensitive	16 (57.1)	29.0 (16.8-41.2)	
Resistant	12 (42.9)	16.3 (13.6-19.0)	
CD133 expression			0.033
Negative	15 (51.7)	29.0 (20.9-37.1)	
Positive	14 (48.3)	19.8 (11.7-27.9)	

*Abbreviations:* *TAH* Total abdominal hysterectomy, *BSO* Bilateral salpingo-oophorectomy.

*P <0.0001 for TAH + BSO *vs.* Limited and for TAH + BSO *vs.* Biopsy, *P* = 0.025 for Limited *vs.* Biopsy.

†*P* = 0.683 for Chemotherapy *vs.* None, *P* = 0.956 for Chemotherapy vs. Chemotherapy + Radiotherapy, *P* = 0.918 for Chemotherapy + Radiotherapy vs. None.

**Figure 2:**
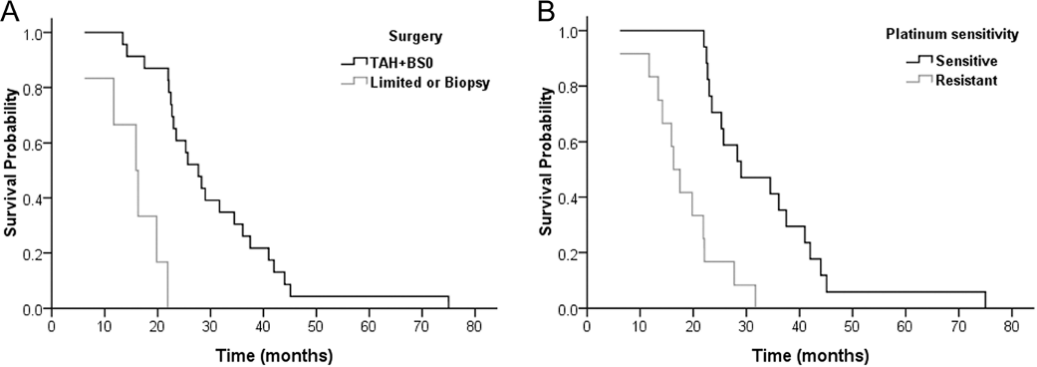
**Kaplan–Meier curves of cumulative central nervous system (CNS)-metastases-free survival (time to CNS metastases) in 29 patients with CNS metastases from ovarian cancer. (A)** Total abdominal hysterectomy + bilateral salpingo-oophorectomy (TAH + BSO) *vs.* limited surgery or biopsy. Result of multivariate Cox regression; HR, 5.91; 95% CI, 1.02-34.24; *P* = 0.047. **(B)** Platinum-resistant *vs.* platinum-sensitive disease. Result of multivariate Cox regression; HR, 5.41; 95% CI, 1.63-17.99; P = 0.006.

Multivariate analysis showed that a smaller extent of surgical resection (HR, 5.91; 95% CI, 1.02-34.24; *P* = 0.047) and platinum resistance (HR, 5.41; 95% CI, 1.63-17.99; *P* = 0.006) were independent predictors for a shorter time to the diagnosis of CNS metastases (Table 7, Figure 2).

**Table 7:** **Multivariate Cox proportional hazards regression for time to CNS metastases**

Variable	HR (95% CI)	***P***value
Age > = 60 yrs	1.38 (0.50-3.82)	0.536
FIGO stage: 3,4 *vs.* 1,2	3.65 (0.38-35.21)	0.263
Surgical resection: TAH + BSO *vs.* Limited and Biopsy	5.91 (1.02-34.24)	0.047
Presence of lymph node metastasis	2.65 (0.91-7.71)	0.074
Platinum resistance	5.41 (1.63-17.99)	0.006
CD133 expression	1.24 (0.46-3.34)	0.665

*Abbreviations:* *TAH* Total abdominal hysterectomy, *BSO* Bilateral salpingo-oophorectomy.

### Prognostic factors associated with OS after the diagnosis of CNS metastases

The median OS since the primary EOC was 3.35 years (95% CI, 2.75-3.95 years), with 1-, 3-, and 5-year survival probabilities being 96.6%, 62.1%, and 17.2%, respectively. The median OS since CNS metastases was 13.2 months (95% CI, 6.9-19.5 months), with 6-month, 1-year, and 3-year survival probabilities being 82.8%, 55.2%, and 9.2%, respectively. Twenty-seven of 29 (93.1%) patients died within the follow-up period. Of the 19 patients whose treatment included neurosurgery, the median OS since the diagnosis of CNS metastases was 17.0 months (95% CI, 11.6-22.4 months), which was significantly longer than that of the 10 patients treated without neurosurgery (8.0 months, 95% CI, 4.5-16.2 months, *P* = 0.004).

Univariate analysis showed significant association between OS and the following parameters: platinum sensitivity, CD133 expression in primary EOC, number of CNS metastases, treatment strategies for CNS metastases, and CD133 expression in CNS metastases (Table 8). Shorter time to CNS metastases diagnosis was not associated with decreased survival.

**Table 8:** **Univariate analysis for predictors of OS since CNS metastases**

Variables	No. of patients (%)	OS since CNS metastases, mo (95% CI)	***P***value
Age (yrs)			0.150
<60	16 (55.2)	15.3 (3.7-26.9)	
> = 60	13 (44.8)	9.4 (4.9-13.9)	
FIGO stage			0.069
1,2	3 (10.3)	32.3 (16.9-47.7)	
3,4	26 (89.7)	11.7 (9.5-13.9)	
Pathology of primary cancer			0.824
Serous	16 (55.2)	11.7 (9.9-13.5)	
Non-serous	13 (44.8)	15.3 (6.1-24.5)	
Histological grade			0.391
1,2	5 (21.7)	22.7 (3.7-41.7)	
3,	18 (78.3)	11.7 (9.6-13.8)	
Extent of surgical resection			0.211*
TAH + BSO	23 (79.3)	13.2 (7.6-18.8)	
Limited	5 (17.2)	16.2 (4.5-19.3)	
Biopsy	1 (3.4)	1.0	
Lymph node metastasis			0.372
No	11 (37.9)	20.4 (8.4-32.4)	
Yes	18 (62.1)	11.7 (9.4-14.0)	
Ascites at the time of primary surgery			0.421
No	10 (34.5)	15.3 (1.7-28.9)	
Yes	19 (65.5)	12.5 (9.9-15.1)	
Adjuvant therapy			0.071†
Chemotherapy	25 (86.2)	15.3 (8.0-22.6)	
Chemotherapy + Radiotherapy	3 (10.3)	7.0 (1.5-12.4)	
None	1 (3.4)	32.3	
Platinum sensitivity			0.033
Sensitive	16 (57.1)	15.3 (0.5-30.1)	
Resistant	12 (42.9)	9.4 (0.1-18.9)	
CD133 expression in primary cancer			0.001
Negative	15 (51.7)	25.5 (10.4-40.6)	
VPositive	14 (48.3)	9.4 (2.4-16.4)	
KPS score			0.412
> = 80	14 (48.3)	13.2 (1.0-29.2)	
<80	15 (51.7)	12.5 (1.1-23.9)	
No. of metastases			0.027
Single	11 (37.9)	25.5 (12.6-38.4)	
Multiple	18 (62.1)	9.4 (4.4-14.4)	
Prior cancer relapse before the diagnosis of CNS metastasis			0.053
No	24 (82.8)	15.3 (6.4-24.2)	
Yes	5 (17.2)	9.4 (3.3-21.2)	
Presence of extracranial disease			0.458
No	16 (55.2)	13.2 (3.1-23.3)	
Yes	13 (44.8)	12.5 (5.4-19.6)	
Treatment of CNS metastases			0.027‡
Steroids	2 (6.9)	1.0 (0–5.292)	
Unimodal (WBRT or Neurosurgery)	9 (31.0)	8.0 (3.0-13.0)	
Multimodal including WBRT (Neurosurgery + WBRT +/− chemotherapy)	10 (34.5)	13.2 (7.5-18.9)	
Multimodal including SRS (SRS +/− neurosurgery +/− chemotherapy)	8 (27.6)	27.3 (15.1-39.5)	
CD133 expression in CNS metastases			0.005§
Negative	3 (15.8)	42.1 (31.4-46.2)	
Positive single cell	4 (21.1)	27.3 (25.1-29.6)	
Positive cluster	12 (63.2)	11.6 (8.5-14.7)	
Time to CNS metastases (mo)			0.122
<23.5	14 (48.3)	7.6 (0.78-20.25)	
> = 23.5	15 (51.7)	15.3 (1.41-29.19)	

*Abbreviations:* *TAH* Total abdominal hysterectomy, *BSO* Bilateral salpingo-oophorectomy, *KPS* Karnofsky performance status, *WBRT* Whole-brain radiation therapy, *SRS* Stereotactic radiosurgery.

*P = 0.211 for TAH + BSO *vs.* Limited, *P* <0.0001 for TAH + BSO *vs.* Biopsy, *P* = 0.025 for Limited *vs.* Biopsy.

†*P* = 0.071 for Chemotherapy *vs.* Chemotherapy + Radiotherapy, *P* = 0.412 for Chemotherapy *vs.* None, *P* = 0.182 for Chemotherapy + Radiotherapy *vs.* None.

‡*P* = 0.027 for Steroids *vs.* Unimodal, *P* <0.0001 for Steroids *vs.* Multimodal including WBRT, *P* = 0.001 for Steroids *vs.* Multimodal including SRS, *P* = 0.022 for Unimodal *vs.* Multimodal including WBRT, *P* <0.0001 for Unimodal *vs.* Multimodal including SRS, *P* = 0.020 for Multimodal including WBRT *vs.* Multimodal including SRS.

§*P* = 0.005 for negative *vs.* positive cluster , *P* = 0.023 for positive single cell *vs.* positive cluster, *P* = 0.075 for negative *vs.* positive single cell.

**Figure 3:**
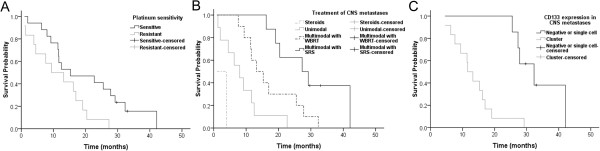
**Kaplan–Meier curves of cumulative overall survival after the diagnosis of central nervous system (CNS) metastases in 29 patients with CNS metastases from ovarian cancer. (A)** Platinum-resistant *vs.* platinum-sensitive disease. Result of multivariate Cox regression; HR, 5.13; 95% CI, 1.28-20.57; P = 0.021. **(B)** Multimodal therapy including stereotactic radiosurgery (SRS) vs. multimodal therapy including whole-brain radiation therapy (WBRT) *vs.* unimodal therapy with WBRT or neurosurgery *vs.* steroids. Result of multivariate Cox regression; multimodal therapy including SRS vs. others: HR, 0.12; 95% CI, 0.03-0.55; *P* = 0.007. **(C)** CD133 cluster formation *vs.* CD133 negative or single cell. Result of multivariate Cox regression; HR, 12.08; 95% CI, 1.55-94.16; P = 0.017.

Multivariate Cox proportional hazards model including variables with *P* <0.05 in the univariate analysis were analyzed to evaluate independent predictors of OS. Platinum resistance (HR, 5.13; 95% CI, 1.28-20.57; *P* = 0.021), multimodal therapy incorporating SRS for CNS metastases (HR, 0.12; 95% CI, 0.03-0.55; *P* = 0.007), and CD133 cluster formation in CNS metastases (HR, 12.08; 95% CI, 1.55-94.16; *P* = 0.017) were found to influence OS significantly and independently (Table 9, Figure 3).

**Table 9:** **Multivariate Cox proportional hazards regression for OS after CNS metastases**

Variable	HR (95% CI)	***P***value
Platinum resistance	5.13 (1.28-20.57)	0.021
CD133 expression in primary ovarian cancer	2.82 (0.66-12.17)	0.163
Multiple CNS metastases	1.11 (0.20-6.12)	0.921
Multimodal therapy including SRS (SRS +/− neurosurgery +/− chemotherapy) for CNS metastases	0.12 (0.03-0.55)	0.007
CD133 cluster formation in CNS metastases	12.08 (1.55-94.16)	0.017

*Abbreviations:* *TAH* Total abdominal hysterectomy, *BSO* Bilateral salpingo-oophorectomy, *KPS* Karnofsky performance status, *WBRT* Whole-brain radiation therapy, *SRS* Stereotactic radiosurgery.

## Discussion

CNS metastases represent a late manifestation of EOC and are associated with extremely poor prognosis regardless of the treatment [1, 4]. One of the main findings of this study is that the extent of surgical resection and platinum sensitivity of primary EOC were the independent risk predictors for time to CNS metastases. This is in keeping with previous observations that the size of residual tumor after surgery is one of the most important prognostic factors for survival of advanced EOC [22, 23]. Our results reemphasize that all attempts should be made to achieve complete cytoreduction or optimal (<1 cm) residual disease in order to prolong the survival of EOC patients and delay disease progression and metastasis.

Given that the current guidelines for the management of EOC should be individualized according to the patient status, gynecologists must balance the risk of rapid metastases against the costs and adverse effects that accompany aggressive interventions. It has been shown by Sehouli et al. [7] and confirmed by our study that platinum sensitivity is an independent prognostic factor for a favorable outcome in patients with CNS metastases from EOC. In addition, our results showed a detrimental impact of platinum resistance on the time to the development of CNS metastases. Taken together, these findings suggest that patients who are unable to achieve optimal cytoreduction and/or who present with platinum resistance might benefit from more aggressive treatment intended to better control the primary disease, and have a possible delay of CNS metastases. Physicians should also pay more attention to the presence of neurological symptoms in this group of patients and arrange CNS imaging for early diagnosis and prompt treatment of metastases. In addition, having a biomarker, which is associated with the metastatic disease would allow this population of patients to be screened appropriately. Our results indicate a positive association between CD133+ expression in primary tumor and increased risk of CNS metastases, and thus hold promise for further validation of the application of this molecule as a biomarker in disease monitoring and management.

Regarding the prognostic factors for CNS metastases from EOC, the findings that platinum sensitivity [7] and multimodal treatment [9, 12] have a positive impact on OS are supported by our results. Moreover, we for the first time compared the expression of CD133, a putative CSC marker, in both the primary EOC and its corresponding CNS metastases, and described its predictive role for CNS metastases.

The fraction of CD133+ cells are enriched in several kinds of solid tumors including the ovarian cancer, which are presented with enhanced resistance to platinum-based chemotherapy [14, 24]. To this end, patients with CD133+ tumor cells are more likely to experience platinum resistance (also confirmed by our results) and thus a less than satisfactory outcome of the primary cancer management. In addition, CD133+ ovarian cancer cells display a potential of CSCs [14], which may be associated with more aggressive tumor growth and poor prognosis in ovarian cancer patients [17]. Several recent studies have also demonstrated CD133 as a metastasis-related molecule. Specifically CD133 + CXCR4+ cancer cells had a high metastatic capacity in liver metastases of colorectal tumors [25], metastatic pancreatic cancers [26], while overexpression of CD133, CD44v6 and human tissue factor was associated with pancreatic carcinoma metastasis [27]. In agreement with these, we found that CD133+ expression in primary EOC was the only independent risk factor for CNS metastases. The fact that all other clinicopathologic parameters were not risk factors indicated that without assessment of the molecular behavior of the primary disease, it may be hard to identify recurrent EOC patients with high risk of CNS metastases who need close observation.

Our data do not support the role of CD133 expression in primary EOC as a significant predictor of the time to CNS metastases diagnosis or subsequent survival of patients with CNS metastases. This is despite CD133 association with platinum sensitivity, a predictor for both CNS-metastases-free survival and OS and could be attributed to a possible bias due to a small number of patients. Furthermore, CD133 may influence patient survival independently of its association with platinum sensitivity, as was shown in a colon cancer study, which found a downregulated CD133 expression in tumor epithelial cells after metastatic transition [28]. A transformation of primary cancer with CD133+ cells into metastasis consists of CD133- cells was observed, indicating that CD133- cells are also potent in tumor initiation [28]. On the other hand, when comparing EOC patients with and without CNS metastases, the overall survival was significantly better in CD133- expressing cases. Thus CD133 may be considered a hallmark of malignancy of primary disease with respect to CNS metastasis. However, its biological function might not be the only rate-limiting step considering that multiple molecular events are known to regulate the process of tumor metastasis [29].

We were able to show that CD133 cluster formation in CNS metastases could serve as a prognostic factor for OS and that CD133 clusters were significantly associated with prior CD133+ expression in primary EOC. However, the CD133+ expression was greater in the metastatic tissue. It is not inconceivable that the microenvironment for CSCs represented by CD133+ staining may be completely different in the CNS compared to the ovary affecting the proliferative or self-renewal potential of CSCs. Also, it has been demonstrated that a significant number of genes are differentially expressed in metastatic disease compared to primary ovarian cancer [30]. To better understand the role of a “stemness” marker of CD133 in the progression and metastasis of primary tumor, further studies are warranted preferably by analyzing a panel of potential “stemness” markers such as CD44, ALDH, EpCAM as well as CD133 in the future [14, 31].

In most cases with CNS metastases from EOC, multimodal treatment approaches have been proven to greatly increase the therapeutic potential and significantly prolong the OS compared to unimodal approaches [3, 6, 8, 9, 12, 32]. Though the optimal combination of modalities remains a matter of investigation, studies with a large sample size as well as meta-analyses showed that a combination of WBRT and neurosurgery with or without chemotherapy was most commonly applied with promising results. SRS has come into focus for the treatment of CNS metastases in recent years, as it is a noninvasive modality that provides good local control [33, 34]. In addition, SRS is capable of treating lesions inaccessible to neurosurgery with an equivalent efficiency, which accounts for 50% of single CNS metastases [1]. Previous study on monotherapy with SRS has shown a remarkable increase in survival compared to WBRT (29 *vs.* 6 months) [35]. In the current study, improved survival was observed in patients treated with multimodal approach including SRS, compared to steroids, WBRT or neurosurgery alone, and multimodal approach including WBRT (27.3 *vs.* 1.0 *vs.* 8.0 vs. 13.2 months, *P* <0.05 in all instances, Figure 3B). In agreement with Kim et al. [11], we also found that the treatment modality including SRS was also the most important independent prognostic factor for CNS metastases from EOC.

This study has several limitations. Although we found that CD133 expression was a risk factor for the development of CNS metastases and that non-optimal cytoreduction and platinum resistance were risk factors for shorter time to the diagnosis of CNS metastases, identification of other potential markers such as transcriptional factors [36–39] and immune factors [40–42] in primary EOC to indicate future cisplatin-refractory CNS-metastasis in EOC patients was outside the scope of this study. A genome-wide transcription analysis may identify other candidate molecules that have different expression levels in EOC patients with and without CNS metastases. Since this was a retrospective study, complete data were not available for all patients. Furthermore, our data represents the experience of a single institution and may not be fully generalizable. Since the number of patients with CNS metastases is small in any single institution, multicenter studies are needed to increase the statistical power. Finally, because CNS metastases in EOC patients often represent as a palliative situation, the quality-of-life should also be considered as an important endpoint.

## Conclusions

In conclusion, our results show a significant association between positive expression of CD133 in primary EOC and an increased risk of CNS metastases. Higher platinum sensitivity is associated with a longer time to develop CNS metastases and a better prognosis of CNS metastases. Absence of CD133 clusters in metastatic tissue and use of multimodal treatment including SRS are associated with prolonged survival. Further investigation is warranted to elucidate the true nature of the association between platinum sensitivity, CD133 expression, and disease outcomes.

## Abbreviations

CNS: Central nervous system
EOC: Epithelial ovarian cancer
SRS: Stereotactic radiosurgery
BBB: Blood–brain barrier
CSCs: Cancer stem cells
OS: Overall survival
WBRT: Whole-brain radiation therapy
FIGO: International federation of gynecology and obstetrics
ALDH: Aldehyde dehydrogenase
EpCAM: Epithelial cell adhesion molecule.
